# Evaluation of Immunostimulatory Effects of Bacterial Lysate Proteins on THP-1 Macrophages: Pro-inflammatory Cytokine Response and Proteomic Profiling

**DOI:** 10.1155/jimr/2289241

**Published:** 2025-04-25

**Authors:** Md. Mijanur Rahman, Asma Talukder, Md. Sifat Rahi, Plabon Kumar Das, I. Darren Grice, Glen C. Ulett, Ming Q. Wei

**Affiliations:** ^1^School of Pharmacy and Medical Sciences, Griffith University, Gold Coast, Queensland, Australia; ^2^Department of Microbiology, Noakhali Science and Technology University, Noakhali, Chittagong, Bangladesh; ^3^Institute for Biomedicine and Glycomics, Griffith University, Gold Coast, Queensland, Australia

**Keywords:** bacterial lysate proteins, cancer, immunostimulants, infectious diseases, pro-inflammatory cytokines

## Abstract

Bacterial lysate proteins (BLPs) serve as potential immunostimulants, recognized by pattern recognition receptors (PRRs) on immune cells, eliciting a robust immune response. In this study, THP-1 macrophages were treated with varying doses of BLPs derived from *Streptococcus pyogenes* (SP), *Streptococcus agalactiae* (SA), and *Serratia marcescens* (SM). The results showed significant increases (*p*  < 0.05) in pro-inflammatory cytokines such as TNF-*α*, IL-1*β*, IL-6, IL-12, granulocyte macrophage-colony stimulating factor (GM-CSF), eotaxin, and macrophage inflammatory protein (MIP)-1*α*, except for 5 µg of all BLPs for TNF-*α* and eotaxin, and 5 µg of SP for IL-12 production. No significant differences were found between the corresponding doses of SP and SA or SP and SM, except for GM-CSF in all doses, while SA and SM only showed a difference at the 5 µg dose for GM-CSF. Furthermore, there were no significant differences between the 10 and 20 µg doses of all BLPs, indicating that doses higher than 10 µg do not significantly enhance the pro-inflammatory response. Combination doses of SP + SM and SA + SM did not show significant differences, except for IL-1*β*, suggesting no synergistic effect. Cytotoxicity was observed to increase with higher BLP concentrations in a dose-dependent manner, with combinations of SP + SM and SA + SM exhibiting greater cytotoxicity than the individual BLPs. Proteomic analysis of BLPs identified immunostimulatory proteins, including heat shock proteins (HSPs; ClpB, DnaK, and GroEL), metabolic enzymes (glyceraldehyde 3-phosphate dehydrogenase (GAPDH), enolase, and arginine deiminase (ADI)), and surface and secreted proteins (ESAT-6-like protein, CRISPR-associated endonuclease Cas9, OmpA, porin OmpC, and serralysin), which are involved in immune modulation, bacterial clearance, and immune evasion. This study underscores the potential of bacterial proteins as vaccine adjuvants or supplementary therapies; however, further research is essential to find a balance between immune activation and inflammation reduction to develop safer and more effective immunostimulants.

## 1. Introduction

Bacterial-origin immunostimulants are noteworthy for their capacity to directly stimulate or augment the host's immune response. Numerous bacterial compounds are reported to elicit a vigorous immune response by promoting affinity maturation of the antibody response, augmenting serum antibody titers, and facilitating immunoglobulin class switching [1–3]. Bacterial lysates (BLs) contain antigen-rich cell fragments, pathogen-associated molecular patterns (PAMPs), and various immunostimulants, which enhance the immune system's ability to identify and fight infections [4–8]. Claims suggest that these preparations induce both local and systemic immunomodulation; however, clinical trials have yielded inconsistent results. BLs are extensively utilized in therapeutic contexts, yet the effects on the immune system remain only partially understood [9–11].

The host's innate immune system can identify PAMPs via pattern recognition receptors (PRRs), triggering innate and subsequent adaptive immune responses. PRRs, expressed by immune cells such as macrophages, dendritic cells (DCs), natural killer (NK) cells, monocytes, neutrophils, mast cells, basophils, and eosinophils, recognize PAMPs on the cell surface, in endocytic vesicles, and within the cytoplasm [12–16]. Innate immune cells lack specific antigen recognition receptors, in contrast to T and B cells, which exhibit high specificity [17]. PAMPs can be used as vaccine adjuvants that modulate antigen-specific immune responses in viral infections and cancer treatment, with Toll-like receptor (TLR) ligands emerging as particularly promising candidates for modern vaccine development [3]. Despite the successful development of several PAMP adjuvants, they remain insufficient overall, with many remaining in the preclinical investigation phase [18–20].

Since the 1950s, biological agents have been employed to decrease the frequency of recurrent respiratory tract infections in both children and adults [21]. Commercially available BLs, such as OM-85, Lantigen B, Ismigen, and LW 50020, have demonstrated effectiveness in both children and adults for the treatment of respiratory tract infections, chronic obstructive pulmonary disease, rhinitis, and rhinosinusitis, albeit with varying degrees of success [10]. Additionally, OM-89, Uromune, Urovac, Urivac, and ExPEC4V demonstrated therapeutic efficacy in managing urinary tract infections in adults, particularly women [10]. BL–based therapeutics demonstrate safety, tolerability, and minimal side effects, positioning them as a viable alternative for controlling infectious diseases [10].

In 1891, William Coley developed “Coley toxins,” a preparation consisting of heat-killed *Streptococcus pyogenes* (SP) and *Serratia marcescens* (SM). Patients diagnosed with sarcomas, lymphomas, melanomas, carcinomas, and myelomas have undergone treatment with varying degrees of success [8, 22, 23]. Subsequently, various immunotherapies based on BLs or extracts were developed, such as intravesical Bacillus Calmette–Guerin (BCG) for in situ bladder carcinoma [24–26], heat-killed *Mycobacterium indicus pranii* for non-small cell lung cancers (NSCLCs) and invasive bladder cancer [27, 28], and heat-killed whole-cell *M. obuense* for melanoma patients [29]. Pro-inflammatory cytokines such as IL-1, IL-6, IL-12, and TNF-*α* have the potential to activate bacterial antigen-specific adaptive responses, which may enhance tumor antigen recognition and response by tumor-specific type 1 helper (Th1) and cytotoxic T cells [30, 31].

PAMPs and PAMP–based compounds are currently under investigation as agonists for TLRs (TLR2, TLR4, TLR5, TLR7, TLR8, or TLR9) in the context of treating infectious diseases and cancer [19, 20, 32]. 3-O-Desacyl-40-monophosphoryl lipid A (MPL), derived from the lipid A component of lipopolysaccharide (LPS) obtained from *Salmonella minnesota*, retains the TLR4 agonist activity while eliminating the toxicity associated with the original molecule. It was the first such compound approved for human use. MPL is an ingredient in the human papillomavirus vaccine (Cervarix) and the hepatitis B vaccine (Fendrix). CpG oligodinucleotides, dsRNA, and flagellin represent three notable PAMP–based adjuvants that have been investigated in recent studies [3, 33]. Imiquimod (Aldara, 3M Pharmaceuticals), an imidazoquinoline analog of ssRNA, is a highly effective medication approved for treating actinic keratosis, external genital warts, and basal cell carcinoma. It operates through a TLR7-dependent mechanism to induce the production of pro-inflammatory cytokines [34]. OK-432 is a bacterial immunostimulant obtained from SP, frequently utilized in cancer therapy and the treatment of lymphangiomas [35]. It enhances the production of Th1 cytokines, including IFN-*γ* and IL-12, facilitating a shift in the immune response towards a Th1-dominant state. It activates key immune cells, including macrophages, polymorphonuclear cells, NK cells, and CD4+ T cells [36–38]. The effectiveness is particularly evident in reducing cysts and enhancing antitumor immunity, notably in pancreatic cancer, lymphatic malformations, and pediatric tumors. Clinical trials have established its safety and efficacy in addressing tumor and cystic conditions, with significant outcomes observed in the treatment of thyroglossal duct cysts, auricular hematomas, pneumothorax, pediatric solid tumors, and advanced ovarian cancer [39–43].

This study investigates the immunostimulatory effects and proteomic profiling of BL proteins (BLPs) in vitro, specifically, those derived from SP and SM, initially utilized in “Coley toxins,” a cancer therapy introduced over 130 years ago, along with the inclusion of an additional gram-positive bacterium, *Streptococcus agalactiae* (SA). We aimed to elucidate the pro-inflammatory cytokine response in THP-1 macrophage cells following treatment with the BLPs that are protein components of “Coley toxins.” It will establish a foundational understanding for the future development of vaccine adjuvants or adjunct therapies for infectious diseases and cancer.

## 2. Materials and Methods

### 2.1. Bacterial Growth and Lysis

SA (ATCC 1138; A909) and SP (ATCC 12384) were cultured in Todd-Hewitt broth at 37°C for a duration of 5 days independently. SM (ATCC 14756) was cultivated in a peptone–glycerol medium (5 g peptone + 10 mL glycerol)/L at 37°C for 5 days with shaking at 200 rpm. The bacterial cell cultures underwent centrifugation at 10,000 rpm for 20 min at 4°C. The bacterial cell culture pellets were resuspended in phosphate-buffered saline (PBS) and maintained on ice. Sonication was conducted to mechanically lyse bacterial cells utilizing a Branson Sonifier 450, applying four cycles of 15 s on followed by 15 s off. The bacteria were maintained on ice during sonication to safeguard the lysate proteins from thermal degradation. The BL was centrifuged at 10,000 rpm for 20 min at 4°C, and then, the supernatant was collected.

### 2.2. Protein Extraction, Filtration, and Quantification

BLs were combined with nine times their volume of cold acetone at −20°C and subsequently mixed using a vortex mixer. The samples underwent overnight incubation at −20°C and were then centrifuged at 15,000 × *g* for 30 min. The supernatant was discarded, permitting the pellet to dry at room temperature for several minutes. The dried pellet was resuspended in a 50 mM ammonium bicarbonate buffer at pH 8. Resuspended proteins underwent filtration using a 0.22 µM syringe filter. The protein concentration was determined using the bicinchoninic acid (BCA) assay, conducted according to the manufacturer's protocol with the Pierce BCA Protein Assay Kit.

### 2.3. Endotoxin Removal and Quantification

BLPs received treatment to eliminate residual endotoxin utilizing high-capacity endotoxin removal resin columns (Pierce, 88274), following the manufacturer's guidelines (Thermo Scientific). The columns were regenerated using 3.5 mL of 0.2 N NaOH overnight. Following washes with 2 M NaCl, ultrapure water, and endotoxin-free buffer, BLPs were added and incubated at 4°C for 16 h. Proteins were recovered via centrifugation at 500 × *g* for 1 min. Endotoxin levels in BLPs were quantified using the LAL Chromogenic Endotoxin Quantitation Kit (Pierce, Thermo Scientific) following the manufacturer's guidelines. After pre-equilibrating the microplate in a heating block for 10 min at 37°C, 50 µL of the sample was dispensed, and then, 50 µL of LAL reagent was added to each well using a pipette. After adding LAL reagent to the plate wells, the plate was removed from the heating block and gently tapped multiple times to ensure proper mixing. The plate was subsequently covered with a lid and placed back in the heating block for incubation at 37°C for 10 min. Subsequently, 100 µL of chromogenic substrate solution, prewarmed to 37°C, was added to each well, mixed gently, and incubated at 37°C for 6 min. Subsequently, 100 µL of stop reagent (25% acetic acid) was added and mixed gently and the absorbance at 405 nm was measured. Endotoxin concentration is expressed in EU/mL.

### 2.4. Cytokine Assay

THP-1 macrophage cells were utilized to assess the production of pro-inflammatory cytokines following immune stimulation with BLPs. THP-1 monocyte cells were seeded in a 24-well plate at a density of 2 × 10^5^ cells per well in 1 mL of RPMI-1640 (Merck, Germany) supplemented with glutamine and sodium bicarbonate, 10% fetal bovine serum (FBS; ThermoFisher Scientific, Australia), 1% penicillin–streptomycin (100 IU/mL penicillin and 100 μg/mL streptomycin), and 0.05 mM 2-mercaptoethanol. Phorbol 12-myristate 13-acetate (PMA; 100 ng/mL) was added to culture media to induce THP-1 monocytes to differentiate into macrophages. The cells were incubated for 48 h at 37°C in a humidified incubator with 5% CO_2_. The induced macrophages were subsequently washed with PBS and provided with PMA-free complete growth media (RPMI-1640), either with treatments (5, 10, and 20 µg of BLPs suspended in 50 mM ammonium bicarbonate buffer, pH 8) or without treatments (control), along with 1 µg of LPS as a positive control. The cells were then incubated overnight in a humidified incubator at 37°C. Following incubation, the cell culture media was transferred to a sterile microtube and centrifuged at 5000 rpm for 5 min at 4°C. The supernatant was collected and stored at −80°C for subsequent experiments.

The pro-inflammatory cytokine response, such as TNF-*α*, IL-1*β*, IL-6, IL-12, granulocyte macrophage-colony stimulating factor (GM-CSF), eotaxin, and macrophage inflammatory protein (MIP)-1*α* was evaluated using the Bio-Plex Pro Human Cytokine Assay Kit from Bio-Rad Laboratories, Hercules, CA, USA. This assay utilizes Luminex xMAP bead-based multiplex technology, facilitating the concurrent measurement of various cytokines from a single sample. The assay was performed in accordance with the manufacturer's guidelines; briefly, 50 µL of each sample, standard, LPS, and control were added to a 96-well plate containing magnetic beads precoated with capture antibodies. The plate was subsequently incubated by shaking at room temperature for 30 min. Following three washes of the plate with wash buffer, 25 µL of detection antibodies were introduced to each well and incubated for 30 min with shaking. After a further wash step (three times), 50 µL of streptavidin–phycoerythrin was added and incubated for 10 min with agitation. The plate underwent three rounds of rewashing and the beads were resuspended in 125 µL of assay buffer. The plate was analyzed utilizing the Bio-Plex 200 system from Bio-Rad Laboratories, with data assessment conducted through Bio-Plex Manager software. Cytokine concentrations were determined through standard curves established from known concentrations of each cytokine.

### 2.5. Cytotoxicity Assay

Cytotoxicity of the BLPs on THP-1 macrophages was determined using the 3-[4,5-dimethylthiazol-2-yl]-2,5 diphenyl tetrazolium bromide (MTT) Assay Kit (Abcam) following the manufacturer protocol. Briefly, THP-1 monocyte cells were seeded in a 96-well plate at 10,000 cells/well density with PMA (100 ng/mL) in RPMI (as mentioned in cytokine assay) and incubated for 48 h at 37°C with 5% CO_2_. Cell culture media was then discarded and fresh media was added with treatments (5, 10, and 20 µg) of BLP or combinations of BLPs (SP + SM and SA + SM, 1:1) suspended in 50 mM ammonium bicarbonate buffer (pH 8) and incubated again at 37°C with 5% CO_2_ for 24 h, and then, treatment media was discarded. Afterward, serum-free media (50 µL) and MTT (50 μL) reagent were added into each well. Background control wells were then prepared with MTT reagent (50 μL) and serum-free cell culture media (no cells) (50 μL). The plate was incubated at 37°C with 5% CO_2_ for 3 h. After incubation, 150 μL of MTT solvent was added to each well. The plate was wrapped in foil and shaken on an orbital shaker at 450 rpm for 15 min. The absorbance was read at 590 nm using a microplate reader. For data analysis, we averaged the triplicate readings for each sample. The culture medium background was subtracted from assay readings to provide corrected absorbance. The percentage cytotoxicity was calculated with the following equation using corrected absorbance:

  $$\begin{array}{c} \%\text{ Cytotoxicity}=\left[\left(\text{Control absorbance}-\text{sample absorbance}\right)\times 100\right]/\text{ control absorbance}. \end{array}$$

### 2.6. Proteomic Analysis

#### 2.6.1. Preparation of Peptide Pools and Clean-up

A pool of peptides was established by digesting proteins with mass spectrometry (MS)-grade trypsin; 10 µg of trypsin was used to digest 100 µg of proteins extracted in the previous step, keeping the overall volume to 100 µL using 50 mM ammonium bicarbonate buffer (pH 8) and incubated overnight at 37°C. To remove salt and other contaminants, we used 1 cc Sep-Pak tC18 Vac cartridge (50 mg) adhering to the following procedures: Reconstitution: The samples were reconstituted in up to 1 mL of 0.1% trifluoroacetic acid (TFA). Conditioning: 1 mL of conditioning solution (90 parts methanol added with 10 parts 0.1% TFA in water) was passed through the tC18 cartridge packing bed and then two additional times without allowing air to enter the packing material. Equilibration: 2 mL of equilibration/load (0.1% TFA in water) solution has been passed through the tC18 cartridge packing bed without allowing air to enter the cartridge packing. Loading: The sample was loaded into the cartridge by slowly passing, and then, 1 mL of additional equilibration/load solution was passed without allowing air to enter the resin bed. Desalting: 1 mL of desalting solution (0.1% TFA in water) was passed through the pack and repeated twice. Elution: The peptides were eluted by passing 1 mL of elution solution (acetonitrile:0.1% TFA, 1:1), and the eluent was then collected in a 1.5 mL microcentrifuge tube. Drying: The peptide sample was dried in a SpeedVac vacuum concentrator [44].

#### 2.6.2. Liquid Chromatography With MS (LC-MS) and Data Processing

The dried peptide samples were resuspended in 50 µL of MS-grade water and centrifuged for 10 min at 14,000 × *g*. Supernatant (40 µL) was then introduced into a MS vial for proteomic analysis. Peptides were introduced onto a pepmap precolumn (75 µM inner diameter), connected to a Dionex UltiMate 3000 RSLCnano HPLC system (Thermo Fisher Scientific, USA) which was connected to an Orbitrap Fusion MS (Thermo Fisher Scientific) via a Nanospray Flex ion source (Thermo Fisher Scientific, USA). The column temperature was held at 45°C using a column oven [44].

The peptides were separated using a monocap C18 nano-flow column (0.1 mm × 150 mm; GL Sciences, CA, USA) with a binary buffer system comprising 0.1% (*v*/*v*) formic acid (buffer A) and 80% (*v*/*v*) acetonitrile/0.1% (*v*/*v*) formic acid (buffer B). Elution was carried out with a 300 nL/minute flow rate over a 130-min LC run. A preliminary BoxCar-DDA library was created utilizing the offline fractionated peptides. The MS1 scans were obtained within the *m*/*z* range of 350–1650, recorded at a resolution of 120,000, with an AGC target set at 250% and a maximum injection time of 246 ms. The Boxcar segments and their corresponding variable widths were derived from Sinitcyn et al. [45],. The BoxCar scans consisted of 24 segments of varying widths, with three multiplexed targeted SIM scans isolating eight segments each. The MS2 scan segments were the same as the MS1 scans. Precursor ions were isolated with an isolation width of 1.6 and accumulated for a maximum duration of 22 ms, while the normalized AGC target was established at 100%. Fragmentation was induced using stepped high-energy collision dissociation (HCD) at 25%, 35%, and 50% collision energies. The MS2 scans were conducted at a resolution of 15,000 at *m*/*z* 200. Only precursors with charge states ranging from 2 to 10 were selected, while unassigned charge states were excluded. The dynamic exclusion of targeted precursors was established for 60 s. A BoxCar DIA analysis was conducted for each sample without fractionation.

The LC and column configuration were the same as those used in the data-dependent acquisition analysis. The BoxCar MS1 and MS2 segments utilized were identical, maintaining a 1-*m*/*z* overlap between boxes in adjacent scans. BoxCar MS1 scans were recorded at a resolution of 120,000, with the normalized AGC target established at 200% per segment and a maximum injection time of 246 ms. MS2 scans of 24 segments were obtained at a resolution of 30,000 and an AGC target of 2000%, with a maximum injection time of 60 ms. Fragmentation was induced using stepped HCD at 22%, 27%, and 32% at collision energies. All raw datasets produced in this study have been submitted to the ProteomeXchange Consortium, accompanied by the dataset identifier. Raw MS data was processed with Spectronaut version 15.1.210713.50606 (Biognosys AG) [46] utilizing default settings and a spectral library created from offline peptide fractionated BoxCar DDA raw files. A maximum of two missed trypsin cleavages was allowed, with cysteine carbamidomethylation designated as a fixed modification and methionine oxidation as a variable modification [44]. To identify immunostimulatory proteins in BLPs, we explored protein functions through the STRING database (https://string-db.org/) and relevant literature sources.

### 2.7. Data Analysis

The 5-parameter logistic (5-PL) regression model was employed to analyze the cytokine assay data using Bio-Plex Manager software. This model was chosen for its ability to provide a more accurate fit for the standard curves, especially when addressing a wide range of concentrations and asymmetrical data. The coefficient of variation (%CV) was calculated, and the %CV values were used to assess the precision of the assay. The acceptable CV for the assay's reproducibility was established below 20%. Statistical significance was evaluated using one-way ANOVA in GraphPad Prism, followed by Tukey's post hoc test, with a *p*-value of less than 0.05 considered significant.

## 3. Results

### 3.1. Endotoxin Quantification

The LAL assay quantified the endotoxin levels in the BLPs. All three separate BLPs were analyzed, revealing that SP (5 µg: 0.004 EU/mL, 10 µg: 0.006 EU/mL, and 20 µg: 0.013 EU/mL), SA (5 µg: 0.005 EU/mL, 10 µg: 0.008 EU/mL, and 20 µg: 0.018 EU/mL), and SM (5 µg: 0.016 EU/mL, 10 µg: 0.041 EU/mL, and 20 µg: 0.084 EU/mL) exhibited endotoxin levels under 0.1 EU/mL.

### 3.2. Pro-inflammatory Cytokine Response

**Figure 1:**
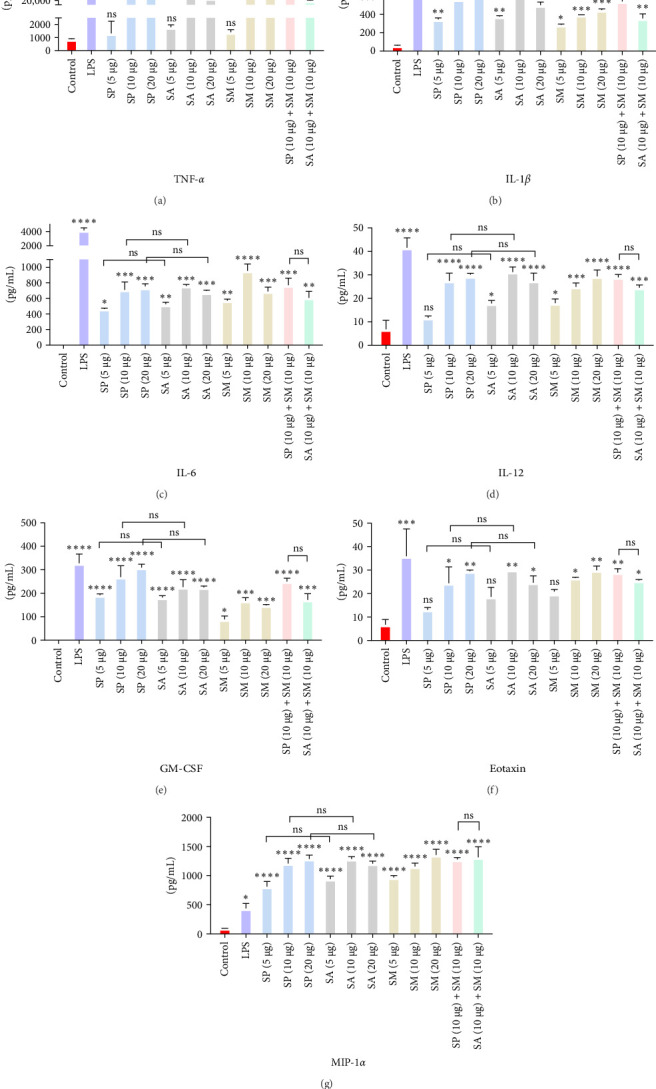
Proinflammatory cytokine response by THP-1 macrophages upon stimulation with different doses of bacterial lysate proteins from *Streptococcus pyogenes* (SP), *Streptococcus agalactiae* (SA), and *Serratia marcescens* (SM). Proinflammatory cytokines, including TNF-*α* (a), IL-1*β* (b), IL-6 (c), IL-12 (d), GM-CSF (e), eotaxin (f), and MIP-1 *α* (g) were produced significantly after treatment compared to without treatment. Here, ns stands for “not significant.” *⁣*^*∗*^*p*  < 0.05, *⁣*^*∗∗*^*p* ≤ 0.01, *⁣*^*∗∗∗*^*p* ≤ 0.001, *⁣*^*∗∗∗∗*^*p* ≤ 0.0001.

Pro-inflammatory cytokines were significantly increased (*p*  < 0.05) by all specified doses of BLPs, except for 5 µg of SP, SA, and SM in TNF-*α* and eotaxin, as well as 5 µg of SP in IL-12. No significant differences were found between the corresponding doses of the two Gram-positive BLPs, SP and SA (5 µg vs. 5 µg, 10 µg vs. 10 µg, and 20 µg vs. 20 µg). Likewise, SP and SM did not show significant differences across doses, except for GM-CSF, while SA and SM only differed at the 5 µg dose for GM-CSF. Furthermore, there were no significant differences in cytokine production between the combination doses (SP + SM vs. SA + SM), except for IL-1*β* (SP + SM: 540.37 pg/mL vs. SA + SM: 356.25 pg/mL; Figure 1 and Table 1). Importantly, no significant differences were noted between the 10 and 20 µg doses of all BLPs in terms of inducing pro-inflammatory cytokines (data only shown for SP vs. SA in Figure 1). The %CV values remained below 20% at all dose points, except for eotaxin, indicating that the assay data were reliable and reproducible (Figure 1, Table 1).

The highest concentrations of cytokines recorded were: TNF-*α* with SM (10 µg) at 29,312.07 pg/mL, IL-1*β* with SP (20 µg) at 624.41 pg/mL, IL-6 with SM (10 µg) at 952.37 pg/mL, IL-12 with SA (10 µg) at 31.27 pg/mL, GM-CSF with SP (20 µg) at 308.99 pg/mL, eotaxin with SA (10 µg) at 30.09 pg/mL, and MIP-1 *α* with SM (20 µg) at 1355.35 pg/mL. However, the highest cytokine levels induced by the aforementioned doses of BLPs were significantly lower for most cytokines compared to LPS (1 µg). Specifically, TNF-*α*, IL-1*β*, IL-6, IL-12, GM-CSF, and eotaxin reached 31.80%, 19.63%, 22.80%, 75.20%, 94.00%, and 83.91% of the levels triggered by LPS, respectively. In contrast, MIP-1*α* was the only cytokine that exceeded LPS-induced levels, reaching 308.52% of the LPS response (Figure 1 and Table 1).

### 3.3. Cytotoxicity of BLPs

**Figure 2:**
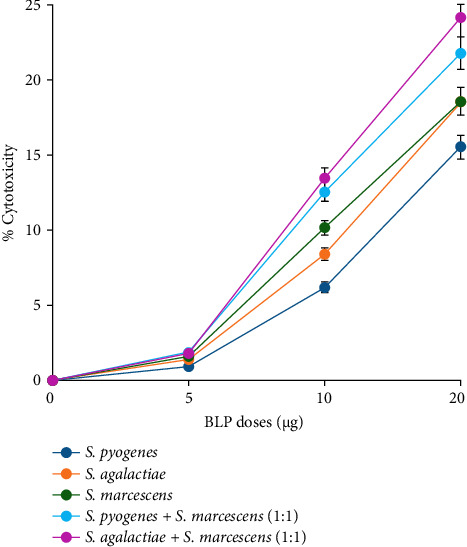
The cytotoxicity of *Streptococcus pyogenes*, *Streptococcus agalactiae*, and *Serratia marcescens* lysate proteins was tested on THP-1 macrophages. All three bacterial lysate proteins showed dose-dependent cytotoxicity.

At a concentration of 5 µg, a slight increase in cytotoxicity was noted: 0.89% for SP, 1.39% for SA, and the highest at 1.60% for SM. In the case of BLP combinations, SP + SM showed a cytotoxicity of 1.87%, while SA + SM had a level of 1.79%. When the dosage was raised to 10 µg, cytotoxicity increased modestly to 6.17% for SP, 8.39% for SA, and 10.13% for SM. The combinations resulted in cytotoxicity levels of 12.56% for SP + SM and 13.45% for SA + SM. At the highest dose of 20 µg, all three BLs demonstrated significant cytotoxicity, with SA and SM presenting similar levels at 18.57%, while SP recorded 15.53%. The combinations exhibited cytotoxicity levels of 21.78% for SP + SM and 24.15% for SA + SM (Figure 2).

### 3.4. Proteomics Analysis

**Figure 3:**
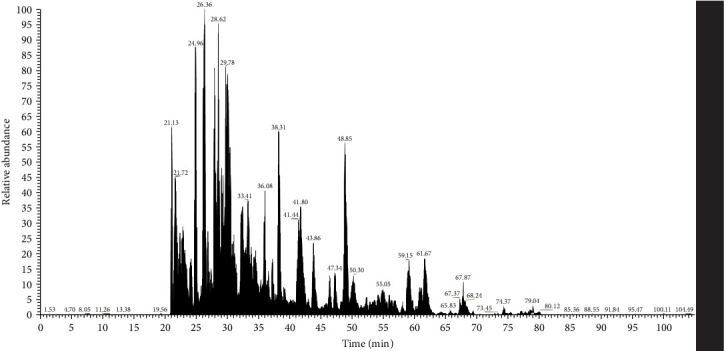
Base peak chromatogram bacterial lysate proteins from *Streptococcus pyogenes* were revealed through LC-MS analysis.

**Figure 4:**
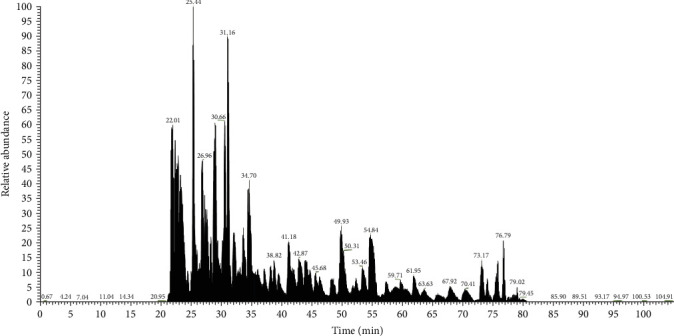
Base peak chromatogram of bacterial lysate proteins from *Serratia marcescens* were revealed through LC-MS analysis.

MS analysis revealed a range of immunostimulatory proteins, including heat shock proteins (HSPs), metabolic enzymes, and surface or secreted proteins in SP, SA, and SM. In HSPs, SM contained ClpB, a chaperone that plays a critical role in protein disaggregation during stress and also enhances the production of pro-inflammatory cytokines and bacterial clearance. Besides, chaperonins DnaK and GroEL were present in all three species, essential for proper protein folding, stimulating robust immune responses, and bolstering adaptive immunity. Metabolic enzymes such as glyceraldehyde 3-phosphate dehydrogenase (GAPDH) and enolase, which are key players in glycolysis, also contribute to immune modulation and bacterial clearance. In addition, both SP and SA produced arginine deiminase (ADI), which helps in immune evasion by lowering extracellular arginine levels, thereby affecting immune responses. Among the surface and secreted proteins, SA also generated ESAT-6-like protein and CRISPR-associated endonuclease Cas9, both of which are significant in immune responses and adaptive immunity. Meanwhile, SM expressed outer membrane proteins (OMPs) OmpA and porin OmpC, along with serralysin, which facilitate nutrient transport, cause tissue damage, and aid in immune evasion (Figures 3and 4 and Table 2).

## 4. Discussion

BLPs provide a robust and versatile method for boosting immune responses by showcasing a wide range of PAMPs that activate PRRs. In contrast to synthetic and recombinant antigens, which often face challenges with limited immunogenicity, BLPs engage various immune pathways, leading to substantial and enduring immune responses. BL's capacity to stimulate TLRs has positioned them as key players in vaccine development and immunotherapy, especially in cancer treatment, where accurate immune modulation is crucial for targeting tumor cells [10, 47]. Furthermore, they have demonstrated potential in fighting infectious diseases by enhancing host defenses and improving mucosal immunity against bacterial and viral pathogens [10]. With their extensive immunostimulatory properties, BLPs emerge as promising candidates for the next generation of vaccines and therapeutic approaches [3, 18].

Cytokines play a crucial role in regulating both innate and adaptive immunity. Consequently, immunostimulatory cytokines, including IL-2, IFN-*γ*, IL-12, and GM-CSF, represent promising candidates for vaccine development [3, 48–50]. Results from this study, as illustrated in Figure 1 and Table 1, indicate that BLPs from SP, SA, and SM significantly enhance the production of various pro-inflammatory cytokines (TNF-*α*, IL-1*β*, IL-6, IL-12, GM-CSF, eotaxin, and MIP-1*α*) in THP-1 macrophages. This suggests a robust immune response, potentially providing insights into inflammation mechanisms and informing therapeutic strategies. However, the production of cytokines triggered by BLPs was notably lower than the positive control LPS, except MIP-1*α*. While LPS is a strong immunostimulator, its high toxicity—especially the risk of causing excessive inflammation and septic shock—along with its limited receptor activation and structural variability create significant hurdles for its application as a positive control in immunomodulation studies involving BLPs. On the other hand, BLPs offer a safer option, as their relatively lower pro-inflammatory activity allows for effective immune activation, while minimizing the risk of excessive cytokine release and systemic inflammation. This characteristic makes BLPs more appropriate for controlled immunomodulation [10, 51–53].

TNF-*α* is crucial for immune function as it activates immune cells and triggers apoptosis in tumor cells, which boosts the body's ability to respond to infections and cancers. The rise in TNF-*α* levels after exposure to BLPs has important implications for understanding inflammatory responses and potential treatment strategies [54–56]. As a key component of innate immunity, TNF-*α* facilitates the clearance of pathogens by activating macrophages, recruiting neutrophils, and promoting inflammation [52, 57, 58]. In addition to its role in immune defense, TNF-*α* connects innate and adaptive immunity by improving antigen presentation and activating T cells [59]. The strong induction of TNF-*α* by these BLPs indicates their potential as immunostimulatory agents, especially as vaccine adjuvants [60].

Moreover, IL-1*β* activates immune cells, promoting inflammation, and tissue repair, which benefits the fight against infections and initiates antitumor responses [61]. This cytokine plays a crucial role in the inflammatory response. SP induces IL-1*β* production via the STING pathway, while SA and SM enhance IL-1*β* production in infection models, which is crucial for their pathogenicity [55, 56].

IL-6 is critical in mediating inflammation and stimulating immune cell activation, particularly during acute response to infections and malignancies. SP, SA, and SM all induce IL-6 production, contributing to their inflammatory properties in various infection models [54, 62, 63].

IL-12 is essential for Th1 cell differentiation and the activation of NK cells, enhancing cell-mediated immunity against intracellular pathogens and tumors [64].

GM-CSF is needed to grow and develop granulocytes and macrophages, which are critical for infection response and tissue repair. Research studies showed that both SP and SA induce GM-CSF production, enhancing the immune response and contributing to their inflammatory properties [65–67].

Eotaxin plays a crucial role in the recruitment of eosinophils to sites of allergic inflammation, contributing positively to specific immune responses. The increase in eotaxin production suggests its potential importance in allergic reactions and asthma [54, 63, 68].

MIP-1*α* is synthesized by a range of cells, particularly macrophages, DCs, and lymphocytes. They are mainly acknowledged for their chemotactic and pro-inflammatory characteristics; however, they also facilitate homeostasis. They facilitate the recruitment of monocytes and macrophages to sites of inflammation, thereby enhancing immune responses and promoting tissue repair [69].

The lack of dose-dependent differences, particularly between the 10 and 20 µg doses across all BLPs, suggests a saturation point has been reached in cytokine induction. This means that increasing the dose beyond 10 µg does not significantly boost the pro-inflammatory response, likely due to the limited availability or occupancy of PRRs on immune cells. This observation is consistent with the threshold effect in immune activation, where higher doses do not lead to a proportional increase in cytokine production once receptor saturation occurs. In addition, the nonsignificant differences between SP and SA at the corresponding doses (5, 10, and 20 µg) indicate that these BLPs may activate the immune system through similar mechanisms, likely involving common PRRs such as TLR2. This similarity suggests that SP and SA could be interchangeable in certain immunotherapeutic contexts, like vaccine adjuvants, where their comparable cytokine induction profiles might produce similar outcomes. However, the significant differences noted with GM-CSF between SP and SM underscore the complexity of cytokine-specific responses and the potential impact of unique bacterial components on specific immune pathways. Furthermore, the absence of significant differences between the combination treatments (SP + SM vs. SA + SM) indicates that the combined effects of these BLPs do not show strong synergistic or antagonistic interactions in cytokine induction. This suggests that the immune pathways activated by SP, SA, and SM either overlap or do not interfere with one another, leading to additive rather than synergistic effects. The notable exception with IL-1*β*, where SP + SM resulted in significantly higher levels than SA + SM, suggests a possible synergistic interaction between SP and SM in activating the inflammasome pathway. This finding calls for further investigation to clarify the underlying mechanisms and explore whether this synergy can be utilized for therapeutic applications.

However, excessive production of cytokines, particularly during a cytokine storm, can lead to serious complications, including intense inflammation and tissue damage [70–72]. This overactive immune response may result in widespread organ failure and is frequently observed in conditions such as sepsis and severe viral infections [73, 74]. The excessive release of cytokines like TNF-*α*, IL-1*β*, and IL-6 can worsen inflammation, creating a harmful feedback loop that overwhelms the body's ability to regulate itself. Therefore, managing cytokine levels is essential to leverage their therapeutic effects while reducing the risk of adverse side effects [75]. Nonetheless, careful modulation of BLPs is vital to enhance immune benefits while reducing the risk of excessive inflammation. Proper regulation of cytokine levels is key to leveraging their therapeutic potential while avoiding harmful side effects [75].

The results of the cytotoxicity experiments demonstrated in Figure 2 indicate a dose-dependent increase in the cytotoxicity of THP-1 macrophages upon treatment with BLPs. Higher concentrations of these proteins demonstrated greater potency in inducing cytotoxic effects in THP-1 macrophages. The combination treatments (SP + SM and SA + SM) showed greater cytotoxicity than the individual BLPs, especially at the 20 µg dose. However, the effects observed were not as high as the anticipated additive values, indicating a less-than-additive interaction instead of true synergy. These findings delineate the necessity for precise dose optimization to strike a balance between immunostimulatory effects and potential cytotoxicity, highlighting the critical need for additional research to improve the safety and effectiveness of BLP–based therapies. Prior studies have shown that BLs can stimulate macrophages through TLRs, leading to the production of pro-inflammatory cytokines and subsequent cell death [76]. The heightened cytotoxicity linked to SM lysate proteins may be ascribed to its unique virulence factors, such as proteases and hemolysins, which are recognized for causing significant harm to host cells. Further research is necessary to identify the primary factors contributing to these effects and to explore the potential therapeutic applications of BLPs in immune response modulation [76–78].

**Figure 5:**
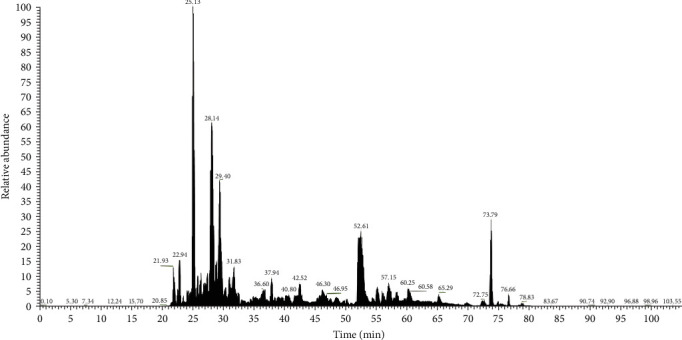
Base peak chromatogram of bacterial lysate proteins from *Streptococcus agalactiae* were revealed through LC-MS analysis.

Proteomic analysis of BLPs (Figures 3–5 and Table 2) identified several immunostimulatory proteins, including HSPs such as ClpB, DnaK, and GroEL, which are essential for bacterial survival and host-pathogen interactions [79]. These proteins not only assist bacteria in responding to stress but also act as immunostimulatory agents that can trigger immune responses in the host [80–83].

ClpB, which belongs to the Hsp100 family and is found in SM, in addition to its chaperone function, plays a role in immune evasion; its capacity to manage stress-induced protein damage boosts bacterial resistance against the host's immune defenses. Notably, ClpB can also function as an immunostimulatory factor by encouraging the release of pro-inflammatory cytokines and activating innate immune receptors, thus, enhancing the host's antimicrobial response [84–86].

DnaK, an important member of the HSP70 family, acts as a molecular chaperone that plays a crucial role in protein folding and stress responses in SP, SA, and SM. Besides, DnaK acts as an immunostimulatory protein by engaging with host immune cells and activating TLR2 and TLR4 signaling pathways [87], leading to the production of IL-1*β* and IL-12, which are essential for the host's immune defense [88]. Additionally, DnaK boosts macrophage phagocytic activity and encourages antibody production, thus, aiding in the clearance of bacteria [89]. Its ability to influence host immune responses through the regulation of apoptosis highlights its dual function in bacterial survival and immune evasion [90]. Importantly, DnaK from SP and SA triggers strong antibody responses, thereby enhancing host immunity [91].

GroEL, found in SP, SA, and SM, a member of the HSP60 family, is crucial for protein folding, serving as a molecular chaperone that helps refold misfolded proteins during times of stress. These proteins are detected by TLR2 and TLR4, which activate pro-inflammatory signaling pathways such as nuclear factor kappa-light-chain-enhancer of activated B cells (NF-*κ*B) and mitogen-activated protein kinase (MAPK) [91, 92], leading to the production of TNF-*α*, IL-6, and IL-12, enhancing innate immune responses and bolstering adaptive immunity [89]. Moreover, GroEL has been shown to facilitate DC maturation, improve antigen presentation, and boost nitric oxide (NO) production in macrophages, thus aiding in the clearance of bacteria [91–93].

Enolase and GAPDH are notable examples of moonlighting proteins that are involved in glycolysis while also interacting with host plasminogen and complement factors, which facilitate bacterial dissemination and immune evasion [94–97]. Enolase interacts with immune cells, triggering the release of TNF-*α* and IL-6 while also enhancing phagocytosis [97, 98]. Moreover, enolase generates robust antibody responses, aiding in bacterial clearance and bolstering adaptive immunity [99–101]. On the contrary, GAPDH promotes the production of IL-1*β*, IL-6, and TNF-*α*, which enhance immune activation and attract immune cells to the site of infection [96, 97]. GAPDH influences complement activation by binding to complement factor H, thus, inhibiting complement-mediated bacterial destruction and further supporting immune evasion [102]. Moreover, GAPDH from these bacteria has been found to activate TLR2, which boosts innate immune responses and aids in bacterial clearance [103]. GAPDH peptides from *Listeria*, *Mycobacterium*, and *Streptococcus* show considerable sequence similarity and provoke strong immune responses in mice. Notably, GAPDH from *Listeria* exhibited the highest effectiveness, offering cross-protection and positioning itself as a promising candidate for a universal vaccine against these pathogens, especially for protecting older adults [104].

ADI, found in SP and SA, facilitates the conversion of L-arginine into citrulline and ammonia as part of the ADI system. This metabolic pathway is vital for bacterial survival in acidic and anaerobic conditions [105]. Beyond its metabolic roles, ADI is crucial for immune evasion by reducing extracellular arginine, an amino acid essential for T-cell proliferation and NO production. By limiting the availability of arginine, ADI hinders T cell responses, diminishes macrophage activation, and lowers NO-mediated bacterial clearance, thereby aiding bacterial persistence within the host [105, 106]. Interestingly, many cancer cells, such as those from hepatocellular carcinoma, melanoma, and certain leukemias, are auxotrophic for arginine, meaning they cannot produce this amino acid and must obtain it from their environment. By depleting arginine, ADI can inhibit cancer cell growth and trigger cell death [107, 108]. The therapeutic promise of pegylated ADI (PegADI), which is chemically modified with polyethylene glycol to enhance its stability and in vivo half-life, has been shown in clinical trials, especially for cancers that rely on external arginine for tumor growth [109].

Surface and secreted proteins—such as ESAT-6-like and Cas proteins found in SA, OMPs like OmpA and porin OmpC, and secreted proteases such as serralysin found in SM—are crucial for immune modulation, bacterial survival, and pathogenesis [63, 110–113]. These proteins assist bacteria in evading host immunity, manipulating inflammatory responses, and establishing infections.

ESAT-6-like proteins are small and highly immunogenic virulence factors found in gram-positive bacteria, such as SA and *M. tuberculosis*. They disrupt host cell membranes by forming pores, which help bacteria escape from phagolysosomes [114, 115]. These proteins act as strong immunostimulants, activating TLR2 to trigger the production of IL-1*β*, TNF-*α*, and IL-6. They also enhance antigen presentation by promoting the maturation of DCs, thereby stimulating adaptive immune responses [114, 116].

Cas proteins found in SA have long been known for their role in adaptive immunity against bacteriophages [117, 118]. However, recent research has uncovered their contributions to bacterial virulence, stress adaptation, and immune modulation [117, 119, 120]. Among these proteins, Cas9 plays a crucial role by regulating the expression of virulence genes, affecting bacterial metabolism, and aiding in immune evasion through changes in surface antigen expression, which allows bacteria to avoid detection by the immune system [119]. Furthermore, Cas proteins help bacteria withstand oxidative stress, enhancing their survival prospects within the host immune cells like macrophages and neutrophils [121, 122].

OMPs play crucial roles as both structural and functional elements of the outer membrane in gram-negative bacteria, such as SM and *Pseudomonas aeruginosa* [123–125]. These proteins are essential for nutrient transport, biofilm formation, adhesion to host tissues, and evasion of the immune system. Acting as PAMPs, OMPs are detected by TLR4 and TLR2, which trigger the activation of NF-*κ*B and type I interferon pathways, both of which are vital for immune responses [126]. Certain OMPs, like OmpA and porin OmpC, help bacteria resist antimicrobial peptides by modifying membrane permeability [126–130].

Serralysin, a zinc-dependent metalloprotease produced by SM, is crucial for immune evasion and causing tissue damage. It achieves this by breaking down extracellular matrix proteins like elastin, fibrinogen, and collagen, which helps the bacteria spread [63, 131–134]. Additionally, serralysin targets complement components (C3 and C5), immunoglobulins (IgG and IgA), and antimicrobial peptides, which reduces innate immune responses and aids in immune evasion [133, 134]. It also stimulates the release of TNF-*α*, IL-8, and IL-1*β*, resulting in excessive inflammation and tissue damage [132]. Its ability to both provoke and suppress immune responses highlights its significance in the virulence of SM.

Finally, understanding the unique features, benefits, and drawbacks of immunostimulatory proteins present in BLPs is essential for improving their design and application. Recent advancements in immunological mechanisms and technology have transformed adjuvant development from random discoveries to a more strategic and rational approach [3]. When incorporating bacterial-derived immunostimulants into vaccines, it is essential to carefully consider safety and regulatory measures, as these substances can provoke strong immune responses. Although these immunostimulants typically have acceptable safety profiles, their activation of PRRs can lead to inflammation if not properly managed. Striking a balance between toxicity and immune activation presents a significant challenge in creating effective adjuvants. Recent progress in understanding immune pathways is paving the way for the development of safer and more effective bacterial-derived immunostimulant molecules [3]. Tumor-specific immunotherapy, a growing field in cancer treatment, encounters obstacles due to the low immunogenicity of tumor antigens and the immune evasion tactics used by tumors. Immunostimulants are becoming increasingly crucial in tumor vaccine strategies, which aim to trigger a type 1-polarized cellular immune response [135]. The findings from this study are essential for creating targeted therapies that utilize BLPs as immune adjuvants or supplementary treatments for infectious diseases and cancers.

However, a significant limitation of our study is that we do not have a detailed understanding of the specific immune pathways that BLPs activate. The assays we conducted do not allow us to distinguish between different types of cell death or immune response mechanisms. Additionally, our research is limited to in vitro models using THP-1 macrophages. In vivo studies are essential to understand better the immunostimulatory effects of BLPs in a physiological context. Future research should focus on identifying specific immune markers, exploring cell death pathways, and carrying out in vivo experiments to shed more light on how BLPs stimulate the immune system and uncover mechanisms to control inflammation.

## 5. Conclusions

Emergency infectious diseases and cancer pose significant challenges to global health, leading to high rates of illness and death. Immunostimulants play a crucial role in developing vaccines, which drives the quest for new materials to boost our immune responses against infections and tumors. Although some bacterial molecules are already in clinical use, there is a pressing need for safer and more effective immunostimulants. This study demonstrates the significant immunostimulatory effects of BLPs obtained from SP, SA, and SM on THP-1 macrophages. The cytokine profile demonstrated substantial elevations in TNF-*α*, IL-1*β*, IL-6, IL-12, GM-CSF, and chemokines, including MIP-1*α*, suggesting a robust potential for immune activation. The cytotoxicity observed at elevated concentrations of BLPs underscores the necessity for meticulous dose optimization. Furthermore, proteomic analysis has identified various immunostimulatory proteins in BLPs, including HSPs, metabolic enzymes, and surface or secreted proteins. Since receptor locations, immune cell targets, and signaling pathways can vary, each PAMP needs to be examined independently. By combining different therapies and adjuvants, we can strengthen both our innate and adaptive immune responses. Future research should focus on achieving a balance between immune activation and the associated risk of excessive inflammation. This study enhances our understanding of the immunomodulatory properties of bacterial-origin proteins and lays the groundwork for developing safer, more effective bacterial immunostimulants, highlighting their potential as vaccine adjuvants or adjunct therapies for infectious diseases and cancer.

## Figures and Tables

**Table 1:** Proinflammatory cytokine (TNF-*α*, IL-1*β*, IL-6, IL-12, GM-CSF, eotaxin, and MIP-1*α*) levels detected in THP-1 macrophages following stimulation with selected doses of lysate proteins from *Streptococcus pyogenes* (SP), *Streptococcus agalactiae* (SA), and *Serratia marcescens* (SM).

Protein doses	Measurement	Proinflammatory cytokines						
		TNF-*α*	IL-1*β*	IL-6	IL-12	GM-CSF	Eotaxin	MIP-1 *α*
SP (5 µg)	Concentration (pg/mL)	1644.54	345.79	467.06	11.775	191.81	13.12	812.43
	%CV	2.45	8.32	5.78	9.14	3.67	7.89	11.23
SP (10 µg)	Concentration (pg/mL)	24,025.74	561.22	711.62	27.57	268.04	24.36	1212.44
	%CV	9.29	8.84	10.07	6.73	16.49	22.89	6.96
SP (20 µg)	Concentration (pg/mL)	28,636.48	624.41	737.59	30.16	308.99	29.55	1289.35
	%CV	2.12	4.74	4.71	1.06	4.04	1.24	4.99
SA (5 µg)	Concentration (pg/mL)	1780.16	375.62	527.86	17.83	181.47	18.62	942.39
	%CV	10.56	5.47	4.22	8.36	2.89	6.54	9.78
SA (10 µg)	Concentration (pg/mL)	28,211.13	594.95	758.66	31.27	227.32	30.09	1288.97
	%CV	0.97	0.16	2.14	4.16	12.49	0	2.89
SA (20 µg)	Concentration (pg/mL)	22,639.44	499.68	699.56	27.57	225.42	24.62	1212.35
	%CV	0.18	4.51	0.78	6.73	2.48	9.56	3.00
SM (5 µg)	Concentration (pg/mL)	1414.46	285.81	572.63	18.02	88.86	19.85	970.03
	%CV	5.34	7.12	13.45	10.58	14.73	5.92	8.65
SM (10 µg)	Concentration (pg/mL)	29,312.07	395.07	952.37	24.97	169.02	26.69	1167.53
	%CV	3.97	0.20	6.37	3.57	6.70	0.90	4.24
SM (20 µg)	Concentration (pg/mL)	26,777.16	445.69	689.60	29.42	148.31	29.98	1355.35
	%CV	2.64	2.30	5.93	5.40	2.20	4.48	7.32
SP + SM (10 + 10 µg)	Concentration (pg/mL)	25,580.56	540.37	771.14	29.05	251.13	29.01	1281.36
	%CV	1.70	5.65	8.07	2.18	4.43	4.18	2.27
SA + SM (10 + 10 µg)	Concentration (pg/mL)	20,182.33	356.25	607.53	24.60	173.16	25.54	1317.84
	%CV	0.72	9.77	10.15	2.40	14.32	1.39	13.74
LPS (positive Control)	Concentration (pg/mL)	92,181.36	3181.26	4176.51	41.59	328.77	35.86	439.28
	%CV	4.65	1.44	3.52	6.90	10.36	24.75	22.64
Control (C)	Concentration (pg/mL)	865.04	59.85	1.94	6.86	0.96	6.70	97.00
	%CV	6.56	5.70	18.64	13.94	3.45	27.17	5.35

Abbreviation: %CV, coefficient of variation.

**Table 2:** Proteomic analysis revealed the presence of several immunostimulatory proteins in the lysate proteins of *Streptococcus pyogenes*, *Streptococcus agalactiae*, and *Serratia marcescens*.

Groups	Bacterial lysate proteins		
	*Streptococcus pyogenes*	*Streptococcus agalactiae*	*Serratia marcescens*
Heat shock proteins (HSPs)	Chaperonin GroEL (groEL) Chaperone protein DnaK (dnaK)	Chaperonin GroEL (groEL) Chaperone protein DnaK (dnaK)	Chaperonin GroEL (groL) Chaperone protein DnaK (dnaK) Chaperone protein ClpB (ClpB)

Metabolic enzymes	Glyceraldehyde-3-phosphate dehydrogenase (naplr) Enolase (eno) Arginine deiminase (arcA)	Glyceraldehyde-3-phosphate dehydrogenase (gap) Enolase (eno) Arginine deiminase (arcA)	Glyceraldehyde-3-phosphate dehydrogenase (gap) Enolase (eno)

Surface and secreted proteins	—	ESAT-6-like protein (SAG1039) CRISPR-associated endonuclease Cas9 (Cas9)	Outer membrane protein A (DMW43_21545) Porin OmpC (OmpC) Serralysin (DMW43_00635)

## Data Availability

The data that support the findings of this study are available from the corresponding author upon reasonable request.

## Nomenclature

%CV: Coefficient of variation
ADI: Arginine deiminase
ANOVA: Analysis of variance
APCs: Antigen-presenting cells
BCG: Bacillus Calmette–Guérin
BLs: Bacterial lysates
BLPs: Bacterial lysate proteins
CD: Cluster of differentiation
CpG: Cytosine-phosphate-guanine
DCs: Dendritic cells
dsRNA: Double-stranded RNA
GAPDH: Glyceraldehyde 3-phosphate dehydrogenase
GM-CSF: Granulocyte-macrophage colony-stimulating factor
HSPs: Heat shock proteins
IFN: Interferon
IL: Interleukin
LAL: Limulus amebocyte lysate
LC-MS: Liquid chromatography-mass spectrometry
LPS: Lipopolysaccharides
MAPK: Mitogen-activated protein kinase
MPL: 3-O-desacyl-4′-monophosphoryl lipid A
MTT: 3-[4,5-dimethylthiazol-2-yl]-2,5 diphenyl tetrazolium bromide
NF-*κ*B: Nuclear factor kappa-light-chain-enhancer of activated B cells
NK: Natural killer
NO: Nitric oxide
NSCLC: Non-small cell lung cancer
Omp: Outer membrane proteins
PAMPs: Pathogen-associated molecular patterns
PBS: Phosphate-buffered saline
PMA: Phorbol 12-myristate 13-acetate
PRRs: Pattern recognition receptors
RPMI: Roswell Park Memorial Institute
SA: *Streptococcus agalactiae*
SM: *Serratia marcescens*
SP: *Streptococcus pyogenes*
ssRNA: Single-stranded RNA
TFA: Trifluoroacetic acid
Th1: Type 1 helper T cell
TLR: Toll-like receptor
TNF-*α*: Tumor necrosis factor-alpha
xMAP: Multi-analyte profiling.
